# The Role of Growth Hormone and Insulin-Like Growth Factor-I in the Liver

**DOI:** 10.3390/ijms18071447

**Published:** 2017-07-05

**Authors:** Yutaka Takahashi

**Affiliations:** Division of Diabetes and Endocrinology, Department of Internal Medicine, Kobe University Graduate School of Medicine, 7-5-2, Kusunoki-cho, Chuo-ku, Kobe 650-0017, Japan; takahash@med.kobe-u.ac.jp; Tel.: +81-78-382-5861; Fax: +81-78-382-2080

**Keywords:** growth hormone, insulin-like growth factor-I, adult growth hormone deficiency, non-alcoholic fatty liver disease, non-alcoholic steatohepatitis, hepatic stellate cells, cirrhosis

## Abstract

Adult growth hormone deficiency (GHD) is characterized by metabolic abnormalities associated with visceral obesity, impaired quality of life, and increased mortality. Patients with adult GHD show increased prevalence of non-alcoholic fatty liver disease (NAFLD)/non-alcoholic steatohepatitis (NASH), and growth hormone (GH) replacement therapy has been shown to improve these conditions. It has also been demonstrated that a decrease in the GH insulin-like growth factor-I (IGF-I) axis is closely associated with the progression of general NAFLD, suggesting a physiological role of these hormones for the maintenance of the liver. NASH histologically demonstrates inflammation, necrosis, and fibrosis, in addition to steatosis (and is a serious disease because it can progress to liver cirrhosis and hepatocellular carcinoma in a subset of cases). While fibrosis determines the prognosis of the patient, efficacious treatment for fibrosis is crucial; however, it has not yet been established. Recent studies have clarified the essential roles of GH and IGF-I in the liver. GH profoundly reduces visceral fat, which plays an important role in the development of NAFLD. Furthermore, GH directly reduces lipogenesis in the hepatocytes. IGF-I induces cellular senescence and inactivates hepatic stellate cells, therefore ameliorating fibrosis. IGF-I treatment has been shown to improve animal models of NASH and cirrhosis, suggesting potential clinical applications of IGF-I in these conditions. In this review, I will focus on the important roles of GH and IGF-I in the liver, their underlying mechanisms, and their potential therapeutic applications.

## 1. Non-Alcoholic Fatty Liver Disease (NAFLD)

Owing to the increasing prevalence of obesity and type 2 diabetes (T2DM), non-alcoholic fatty liver disease (NAFLD) is now recognized as the most common cause of chronic liver disease worldwide [1,2,3]. NAFLD consists of non-alcoholic fatty liver (NAFL) and non-alcoholic steatohepatitis (NASH), and encompasses liver conditions ranging from simple steatosis to cirrhosis [4]. The diagnosis of NASH is based on a histological examination using liver biopsy [5]. Simple steatosis (NAFL) is characterized by fatty deposits in hepatocytes, while in addition to steatosis, NASH is characterized by inflammatory cell infiltration, hepatocyte ballooning, and fibrosis [3]. Importantly, NASH can progress to cirrhosis and hepatocellular carcinoma in a subset of cases [6].

In most cases, NAFLD occurs based on a presence of risk factors, such as metabolic syndrome, obesity, T2DM, mixed hyperlipidemia, hypocholesterolemia (due to familial hypobetalipoproteinemia), and the carriage of risk alleles for selected genetic polymorphisms [7].

NAFLD is a multi-factorial disease resulting from a complex interaction of environmental “hits” and a genetic background. Insulin resistance associated with visceral obesity, lipotoxicity and inflammation, and dysbiosis in the gut play an important role in the development of NAFLD [3]. In these conditions, increased reactive oxygen species (ROS), dysregulated cytokine induction, and inflammation lead to the activation of hepatic stellate cells (HSCs) and result in fibrogenesis [8].

Fibrosis is a histological and biochemical hallmark during the progression to cirrhosis [9]. Recently, fibrosis has been considered as an active biosynthetic process leading to excess deposition of the extracellular matrix (ECM). HSC activation represents a critical event in fibrosis because these cells become the primary source of ECM in the liver upon injury [10]. A large retrospective study demonstrated that liver fibrosis only, with no other histologic features, was associated with long-term outcomes of patients with NAFLD [11]. Therefore, prevention of fibrosis is crucial [9]. Changes in dietary habits and life style have been recommended as standard care for NAFLD, but this behavioral strategy tends to fail in most patients. To date, there have been very few high quality, randomized, blinded, adequately powered, controlled studies of sufficient duration and with adequate histological outcomes. A GLP-1 analogue liraglutide, PPARγ agonist pioglitazone, vitamin E, and FXR agonist obeticholic acid have been proven to be efficacious [3].

## 2. Endocrine Abnormalities and NAFLD

NAFLD is often observed in patients with endocrine disease, particularly in the impairment of hypothalamic-pituitary axes [12]. These aspects also suggest etiological mechanisms underlying the pathogenesis of NAFLD. In particular, hormonal derangements, such as growth hormone (GH) deficiency and Cushing’s syndrome, are associated with an abnormal body fat distribution and impaired cell metabolism. Therefore, these conditions are causally related with the development of NAFLD [13]. Thyroid hormone plays an essential role in metabolism and energy expenditure. An increased risk for NAFLD is observed in patients with hypothyroidism [14]. In Cushing’s syndrome caused by a cortisol excess, it has been reported that 20% of the patients were diagnosed with NAFLD [15]. Dehydroepiandrosterone (DHEA) is an androgen produced by the adrenal gland, and a decreased serum level of DHEA is associated with the progression of fibrosis in patients with NAFLD [16]. Low serum testosterone levels are associated with hepatic steatosis [17]. In obese men with obstructive sleep apnea, testosterone treatment reduced fat deposits without reducing body weight [18]. Antagonizing estrogen action by tamoxifen drives hepatic steatosis [19], and low serum estrogen levels are associated with the presence of hepatic steatosis [20]. Polycystic ovary syndrome (PCO) presents with impaired ovulation, hyperandrogenemia, and insulin resistance, and PCO is associated with obesity, metabolic syndrome, cardiovascular disease, cirrhosis, and liver tumors. Several cross-sectional and case-control studies have consistently demonstrated that the prevalence of NAFLD is remarkably increased in patients with PCO, independent of being overweight/obese and having other coexisting metabolic syndrome features, and that women with this syndrome are more likely to have the more severe forms of NAFLD [21]. These results suggest that the liver is an important metabolic organ that is regulated by various hormones. In addition, the essential roles of GH and IGF-I in the liver have recently been highlighted.

## 3. The Metabolic Action of Growth Hormone (GH) and Pathophysiology in Adult Growth Hormone Deficiency (GHD)

In addition to its effects on linear growth in childhood, GH plays an important role in the regulation of metabolism, body composition, strength, aerobic capacity, and mood, which persists into adult life [22]. IGF-I production is mainly regulated by GH, and both GH and IGF-I have an anabolic effect on skeletal muscle and bone. The profound metabolic effects of GH involve an increase in lipolysis and protein synthesis, and a decrease in hepatic and muscle insulin sensitivity and glucose uptake. GH has a strong lipolytic effect, preferentially on visceral adipose tissue, with a lesser effect on subcutaneous adipose tissue.

Adult GHD is now recognized as a well-defined clinical condition, characterized by an increased visceral adipose tissue mass, reduced muscle strength and energy, adverse lipid profile, impaired quality of life, and increased mortality, mainly due to the increased risk of cardiovascular disease [22,23]. Interestingly, it has been reported that NAFLD is an independent risk factor for cardiovascular disease [24]. GHD in adults most commonly results from pituitary or peri pituitary organic lesions and their treatment [25]. The diagnosis of adult GHD requires GH provocative tests. GH replacement therapy in adults restores most biochemical and functional abnormalities to or toward normal, and is safe and well tolerated.

## 4. NAFLD Is an Important Complication in Adult GHD

Recently, hepatic complications in adult GHD have emerged [26]. It has been reported that a case with panhypopituitarism demonstrated fatty liver, and after GH administration the liver condition improved, suggesting that fatty liver was at least partly attributable to GH deficiency [27]. In Japanese patients with panhypopituitarism, liver dysfunction and hyperlipidemia have been frequently observed in those with adult GHD [28]. In a small case control study, hepatic steatosis was more prevalent in hypopituitary patients with adult GHD than those without GHD, and one of the patients was diagnosed with NASH using a liver biopsy [29]. In patients with hypothalamic and pituitary dysfunction, a rapid development of NAFLD, a high prevalence of NASH and cirrhosis, and increased risk of liver-related death were observed [30]. Given the fact that GH secretion is most frequently impaired in patients with hypothalamic-pituitary organic disease, it seems that NAFLD was attributable to the GH deficiency in these patients. Regarding the transition period, it has been reported that the prevalence of metabolic co-morbidities, including NAFLD, increased after the cessation of GH treatment in adults with childhood-onset GHD, depending on its duration. In a retrospective analysis, the prevalence of NAFLD increased by 29% at a mean age of 30 years [31].

Interestingly, it has been reported that in a case of adult GHD, GH replacement therapy drastically reversed NASH, concomitant with a decrease in inflammation and oxidative stress markers [32]. Nishizawa et al. reported that in 66 Japanese patients with adult GHD, the prevalence of NAFLD was significantly higher compared to age-, gender-, and BMI-matched controls (77% vs. 12%). In addition, at least 21% of the patients were diagnosed with NASH using liver biopsy [33]. GH replacement therapy improved elevated serum liver enzyme concentrations, as well as histological changes concomitant with a reduction in the fibrotic markers. Furthermore, the results of long-term follow up in these patients have been reported. Over a 24-month period, a significant reduction in serum liver enzyme concentrations and in the fibrotic markers was observed in patients with adult GHD receiving GH replacement therapy, compared with the patients without GH replacement therapy [34]. Furthermore, GH replacement therapy also improved liver enzyme concentrations in adult GHD patients with NASH. Additionally, a body weight gain during the treatment retarded the effect of GH, suggesting that treatment for general risk factors, such as obesity, is also important.

To date, reports have demonstrated contrasting results. Gardner et al. [35] reported that NAFLD was equally common in 28 patients with GHD and in 24 age- and BMI-matched control individuals. Meienberg et al. [36] reported that, although an increased tendency (28% vs. 16%) in the prevalence of NAFLD and the intrahepatic lipid content assessed using MRI (1.89 vs. 1.42) in 22 patients with adult GHD (as compared with matched 44 control subjects) was observed, the difference was not significant. Several factors may explain this discrepancy. The most likely explanation relates to the small sample size. In addition, it is well known that ethnicity, age, sex, and body mass index (BMI) are associated with the prevalence of NAFLD [37]. It has been reported that the prevalence of NAFLD is higher in Caucasian than that in Asian population cohorts. Age-dependent increases in the prevalence of NAFLD and NAFLD-related fibrosis have been reported [37]. A higher BMI increases the prevalence of NAFLD. For example, according to the annual health check in Japan, the prevalence of NAFLD increased with BMI: 10–20% in non-obese individuals, 50% in those with a BMI between 25 and 30 kg/m^2^, and 80% in those with a BMI over 30 kg/m^2^ [2].

When comparing the studies of Nishizawa’s, Gardner’s, and Meienberg’s in reporting the prevalence of NAFLD in adult patients with GHD, Nishizawa’s study demonstrated a significant increase in the prevalence of NAFLD in patients with adult GHD but the other studies did not. Both the age and BMI of the patients were higher (age: 48.2 vs. 52.6 vs. 52.2 years, BMI: 25.2 vs. 27.9 vs. 28.5 kg/m^2^) in Gardner’s and Meienberg’s studies, which may be the cause of the discrepancy as the prevalence of NAFLD increases with age and BMI. In Gardner’s study, the waist circumference of the control group was 101 cm, suggesting the presence of visceral obesity, which might have increased the prevalence of NAFLD in the control group. Indeed, the prevalence of NAFLD was as high as 50% in the control group in this study. Moreover, although it was not statistically significant, GH replacement therapy showed a tendency to decrease liver fat content (*p* < 0.07) in Gardner’s study, suggesting that the patient sample size might have been too small to detect the effect of GH replacement therapy.

Among adult GHD patients, Nishizawa et al. reported that increased BMI, visceral adiposity, dyslipidemia, and presented with insulin resistance were related to the presence of NAFLD. These factors are clearly the main characteristics of adult GHD, suggesting that the pathophysiological condition of adult GHD per se increases the risk for NAFLD [33]. Hong et al. reported that the prevalence of NAFLD was increased in patients with hypopituitarism, and the severity in fatty liver was shown to be related to serum GH level [38]. Taken together, this accumulating evidence strongly suggests that a GH deficient state in adults is closely associated with the development of NAFLD/NASH.

In respect of the role of the GH-IGF-I axis in the pathogenesis of general NAFLD/NASH, it has been reported that serum levels of GH, IGF-I, and IGF-binding protein 3 (IGFBP-3) were associated with hepatic steatosis and fibrosis in patients with NAFLD, even in the non-GH-deficient population [39]. Elevated levels of GH, and decreased levels of IGF-I and IGFBP-3, suggesting a presence of GH resistance, were associated with the severity of the disease in patients with NAFLD but not in patients with HCV-related chronic liver disease [40]. Lower serum IGF-I levels were associated with the severity of inflammation, hepatocyte ballooning [41], and fibrosis [42] in patients with NAFLD. These data strongly suggest that the GH–IGF-I axis plays a role in the liver, even under physiological conditions as well as in GHD (Table 1).

## 5. The Underlying Mechanisms

GH generates IGF-I at various target tissues in autocrine and paracrine fashion [43], but most circulating IGF-I is produced in hepatocytes [44,45]. Liver-specific deletion of the GH receptor in mice (GHRLD) resulted in a 90% reduction in serum IGF-I levels [46]. GHRLD mice showed insulin resistance, glucose intolerance, increased free fatty acids, and severe hepatic steatosis, indicating the physiological importance of GH signaling in the liver. In addition, regeneration of hepatocytes was impaired in GHRLD mice, indicating that GH plays an important role in the proliferation and/or anti-apoptotic capacity of hepatocytes [47,48,49]. In the downstream signaling of the GH receptor, liver-specific JAK2-deficient mice (JAK2L) also developed hepatic steatosis [50]. These mice were lean but they demonstrated an increased liver triglyceride content and plasma FFA levels. As an underlying mechanism, a cross between GH-deficient *lit*/*lit* mice and JAK2L mice resulted in reduced plasma FFA levels and hepatic steatosis, suggesting that GH-induced lipolysis in adipose tissue may play a role in the development of hepatic steatosis in this model. Furthermore, mice with a liver-specific signal transducer and transcriptional activator 5 (STAT5)-deficient mice developed hepatic steatosis, glucose intolerance, insulin resistance, late-onset obesity, and impaired liver regeneration [51]. These data indicate the importance of the GHR-JAK2-STAT5 signaling pathway in the liver.

**Figure 1:**
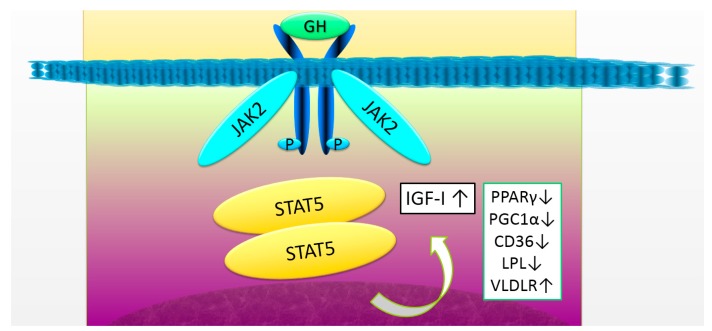
The growth hormone (GH) receptor signaling and target genes in the liver. GH binds to the GH receptor and activates the janus kinase 2 (JAK2)-signal transducer and activator of transcription 5 (STAT5) signaling pathway. Several target genes of STAT5, including insulin-like growth factor-I (IGF-I) and peroxisome proliferator-activated receptor γ (PPARγ), play an essential role in the liver. The decrease in GH receptor signaling induces a decrease in IGF-I expression, resulting in the progression of fibrosis. At the same time, an increase in the expression of PPARγ, peroxisome proliferative activated receptor γ, coactivator-1α(PGC1α), cluster of differentiation 36 (CD36), and lipoprotein lipase (LPL), and a decrease in the expression of very low density lipoprotein receptor (VLDLR), cause the impaired lipid metabolism, leading to steatosis. GH, growth hormone; IGF-I, insulin-like growth factor-I.

There are several underlying mechanisms for GH acting on the hepatocytes (Figure 1). An elevated expression of the peroxisome proliferator-activated receptor γ (PPARγ) and its target gene CD36 in the hepatocytes leads to an increased uptake of FFA. Recent studies with liver-specific STAT5-deficient mice demonstrate that elevated CD36, PPAR γ, and PPAR γ coactivator 1 α/β (PGC1 α/β), along with an increased fatty acid synthesis, lipoprotein lipase, and the VLDL receptor, are associated with hepatic steatosis in these mice [52]. An adult-onset hepatocyte-specific GHR knockdown mouse was developed as a model of hepatic GH resistance after sexual maturation [4]. Hepatic de novo lipogenesis was increased and steatosis developed in the male mice. In addition, liver-specific ablation of GHR in mice leads to increases in lipid uptake, de novo lipogenesis, hyperinsulinemia, and hyperglycemia, accompanied with severe insulin resistance and increased body adiposity and serum lipids. Restoration of IGF-1 through transgene into the hepatocytes improved overall insulin sensitivity and lipid profile, and reduced body adiposity, but was insufficient to protect against steatosis-induced hepatic inflammation and oxidative stress, suggesting a presence of direct action of GH in hepatocytes [53]. Furthermore, Laron syndrome, caused by a loss of function mutations of the *GHR* gene in humans, manifests as NAFLD in adults. Chronic replacement of IGF-I did not influence the NAFLD status, again suggesting that GH has a direct action in the liver, particularly for the prevention of steatosis in hepatocytes (Figure 1) [54].

Regarding the role of IGF-I in the liver, low serum levels of IGF-I have been observed in patients with chronic liver disease, and malnutrition, despite normal or elevated GH secretion [55,56,57], because the hepatocytes produce most of the serum IGF-I, and GH resistance generally occurs in chronic liver disease [45]. It has been considered that IGF-I does not affect hepatocyte function directly because the hepatocytes express few IGF-I receptors (IGF-IR) in a normal condition [58]. However, recent studies have demonstrated an increased expression of IGF-IR in pathological conditions. In chronic hepatitis C, chronic hepatitis B, and liver cirrhosis [59,60], IGF-IR expression was clearly detected in hepatocytes [61] when compared to that of the normal liver, suggesting that IGF-I signaling may play a role in these pathological conditions [58,62].

Recently, accumulating evidence indicates that IGF-I plays an essential role in the liver. A spontaneous dwarf rat, in which GH was deficient, also demonstrated NASH, and IGF-I administration reversed these changes in the liver as well as GH [63]. Although it is unclear whether these effects of IGF-I on the liver resulted from direct or indirect action on hepatocytes, these results clearly indicate that IGF-I plays an important role in the liver in GH-independent mechanisms. One possibility is that IGF-I exerts its effect on hepatocytes by direct action, via an aberrant expression of IGF-IR under pathological conditions, via the insulin receptor (IR), or via hybrid receptors that consist of IR and IGF-IR [64]. Interestingly, Kupffer cells regulating inflammation and HSCs driving fibrosis both express IGF-R, and accumulating evidence suggests that IGF-I exerts influence on these cells and regulates hepatic inflammation and fibrosis.

**Figure 2:**
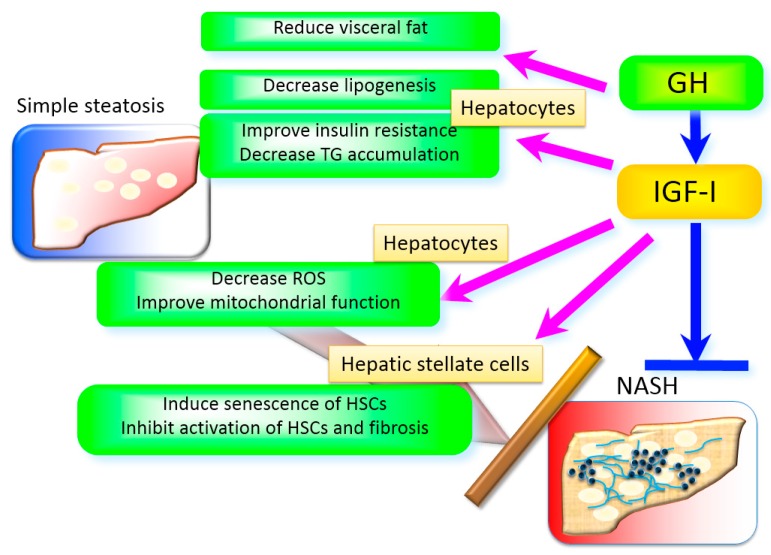
The role of GH and IGF-I in the liver. GH and IGF-I exert their effects in multiple mechanisms. GH reduces visceral fat that plays a pivotal role in the development of non-alcoholic steatohepatitis (NASH). GH directly decreases lipogenesis in the hepatocytes. IGF-I improves insulin resistance, decreases reactive oxygen species (ROS), improves mitochondrial function, and decreases triglyceride accumulation in the hepatocytes. In addition, IGF-I induces senescence and inactivates HSCs and limits fibrosis.

Several underlying mechanisms are responsible for the effects of IGF-I in the liver (Figure 2). It is well known that insulin resistance, oxidative stress, mitochondrial dysfunction, and inflammation play integral roles in the development of NASH [65]. IGF-I improves insulin sensitivity in the muscle and liver. The deletion of the *igf-I* gene in the liver results in insulin resistance [66], indicating that hepatic IGF-I regulates systemic insulin sensitivity. In terms of mitochondrial dysfunction, mitochondrial morphology was severely impaired in the hepatocytes of GH-deficient rats, and IGF-I reversed these changes in the mitochondria [63]. Most ROS is produced in the mitochondria and is closely associated with the impaired function of the mitochondria. It has also been reported that IGF-I improved enhanced oxidative stress in the liver, suggesting that IGF-I regulates mitochondrial function and oxidative stress. In fact, IGF-I improves mitochondrial function in vitro [67] and in vivo [68]. IGF-IR activation improved oxidative stress, mitochondrial dysfunction, and apoptosis in human umbilical vein endothelial cells [67]. IGF-I reduced oxidative mitochondrial damage, improved complex V ATPase activity, and decreased caspase activities [68]. It has also been reported that IGF-I administration improved liver dysfunction and fibrosis in a rat cirrhotic model, and mitochondrial function in aging rats [69]. The double deletion of the *irs-1* and *-2* gene in mice, which are the main molecules downstream of insulin and IGF-I signaling, resulted in a Foxo1 activation (and an increase in its target gene expression, including heme oxygenase-1 (Hmox1), which disrupts complex III and IV of the respiratory chain), and lowered the NAD^+^/NADH ratio and ATP production in the mitochondria [70]. IGF-I may regulate the mitochondrial function via these pathways as well as insulin.

HSCs play a key role in hepatic regeneration and fibrosis progression [71]. Activation of HSCs into the myofibroblast phenotype can be provoked by a range of chronic injuries to the liver, including oxidative stress, inflammatory cytokines, and lipopolysaccharide (LPS) [72]. In cultured HSCs, IGF-I increases proliferation [73] and collagen synthesis [74]. On the other hand, quiescent HSCs do not respond to IGF-I, irrespective of high IGF-IR expression, suggesting that IGF-I action on HSCs is dependent on the stage of differentiation and on the environment [73,75]. In the development of fibrosis, it has been reported that overexpression of IGF-I in HSCs limited their activation, attenuated fibrosis, and accelerated liver regeneration in a carbon tetrachloride (CCl_4_)–treated cirrhotic model [76]. It was observed that the hepatocyte growth factor (HGF) was up-regulated and transforming growth factor β1 (TGFβ1) was down-regulated in the model. Another report also demonstrated that IGF-I stimulated production of HGF, but not TGFβ1, in HSCs [77]. HGF is an essential mitogen for hepatocytes and appears to limit fibrosis in vivo [78].

Recently a novel mechanism, in which IGF-I directly regulates hepatic fibrosis, has been demonstrated. IGF-I administration improves histological changes in NASH and cirrhotic mice models [79]. IGF-IR is strongly expressed in HSCs and IGF-I-induced cellular senescence in HSCs, in vitro and in vivo. Because cellular senescence inactivates the HSCs and limits fibrosis [80], and IGF-I acts on these cells and induces cellular senescence in several kinds of cells [81,82], it has been proposed that IGF-I inactivates HSCs via inducing their senescence. Importantly, in mice lacking the key senescence regulator p53, IGF-I did not induce cellular senescence in HSCs nor show any effects on fibrosis, clearly indicating that IGF-I-induced senescence in HSCs plays an essential role in these effects [79]. Other potential candidates for inducing senescence in HSCs, a matricellular protein CCN1 [83], interleukin-22 [84], and substance P [85] have also been reported. These results suggest that various factors, including IGF-I regulate senescence and the activated status of HSCs and limit fibrosis. Several agents, such as atorvastatin [86] and celecoxib derivative OSU-03012 [87], have been shown to induce senescence and improve fibrosis, indicating that this mechanism may be a novel target for preventing fibrosis.

## 6. The Clinical Applications of GH and IGF-I in NASH and Cirrhosis

To date, several clinical studies have demonstrated a potential application of GH and IGF-I in obesity-related conditions. The strong effect of GH on visceral obesity and dyslipidemia in patients with adult GHD has led to several pilot clinical trials on patients with obesity [88] and liver cirrhosis [89], in whom GH secretion was not impaired. Franco et al. examined the effect of GH on 40 postmenopausal women with visceral obesity [88]. One-year treatment of GH reduced visceral fat mass, increased thigh muscle area, and reduced serum LDL cholesterol levels. Insulin sensitivity was increased in the GH-treatment group. A positive correlation was shown between the changes in the glucose disposal rate and hepatic fat content.

In the study of NASH model choline-methionine fed *db/db* mice, IGF-I administration drastically ameliorated histological changes, along with mice in a DMN-induced cirrhotic model, as well as leading to a biochemical improvement [79]. A limited number of human studies of GH or IGF-I for the treatment of cirrhosis have been conducted without histological examination. It is well known that IGF-I has a strong anabolic action, especially in protein metabolism in muscle tissue, which is generally disturbed in chronic liver disease. Donaghy et al. reported results of a randomized, double-blind, placebo-controlled study of GH treatment in 20 cirrhotic patients. They assessed the GH impact on protein turnover [89]. A relatively high dose of GH (0.25 IU/kg body weight) administration for 7 days significantly increased serum IGF-I levels and improved nitrogen balance in these patients. Interestingly, a prospective randomized study demonstrated that rhGH administration significantly improved the prognosis of the patients with chronic liver failure, suggesting a beneficial effect of GH on a life expectancy [90]. A pilot study showed that IGF-I administration in cirrhotic patients improved serum albumin and energy metabolism after 120 days [91]. Taken together, these data suggest GH or IGF-I may be applicable for the treatment of NASH or cirrhosis with its unique mechanisms, in which especially IGF-I directly inactivates HSCs, concomitant with its anabolic action. It is also suggested that a decrease in IGF-I production in the liver is not only a result of impaired liver function but also plays a key role in the progression of fibrosis.

## 7. Conclusions

In conclusion, accumulating evidence demonstrates that GH and IGF-I play an essential role in the liver. NAFLD/NASH is an important complication in patients with adult GHD. Although further additional human studies are necessary, experimental studies suggest that GH or IGF-I may be applicable for the treatment of NASH or cirrhosis.

## Figures and Tables

**Table 1:** The role of growth hormone (GH) and insulin-like growth factor-I (IGF-I) signaling in the liver.

Receptor/Signaling	Function	References
GH receptor/JAK2/STAT5		
Metabolism	Lipogenesis↓, Steatosis↓	[3,43,46,47,48,49,59]
Fibrosis	Fibrosis↓	[59]
Regeneration	Regeneration↑	[43,44,45]
NAFLD/NASH (human)	improves NAFLD/NASH	[23,25,28,29]
IGF-I receptor		
Metabolism	Steatosis↓	[59,62,75]
Fibrosis	Fibrosis↓↓	[59,65,72,75]
Regeneration	Regeneration↑	[72,73,75]
NAFLD/NASH (human)	improves NASH/cirrhosis	[85,86,87]
